# Signet-Ring Cell Colon Cancer in a Teenager: A Case Report

**DOI:** 10.7759/cureus.12632

**Published:** 2021-01-11

**Authors:** Amy Somerset, David A Edelman, John Webber

**Affiliations:** 1 Surgery, Beaumont Health, Grosse Pointe, USA; 2 Surgery, Wayne State University Detroit Medical Center, Detroit, USA

**Keywords:** colorectal cancer, signet ring cell adenocarcinoma, teenage, metastatic colo-rectal cancer

## Abstract

The majority of colon cancers are diagnosed in the older population, though recent trends have demonstrated an increase in younger patients. Most colon cancers are considered adenocarcinoma. There are multiple histologic subtypes with varying prognoses. Mucinous types such as signet-ring cell carcinoma (SRCC) are more aggressive with poor outcomes. SRCC frequently presents with metastatic disease which contributes to its poor prognosis. It is most commonly diagnosed around age 40. SRCC of colonic origin is very rare and comprises only 1% of colorectal cancers. Rarer still is presentation in the teenaged patient, especially in the absence of any risk factors. We present a case of an 18-year-old male with colonic SRCC. The patient presented initially with vague abdominal discomfort and three weeks later was found to have a near-obstructing right-sided colon mass. He was taken to the operating room and found to have diffuse carcinomatosis. The patient underwent palliative loop ileostomy with plans for subsequent chemotherapy.

## Introduction

Colorectal cancer is the third most common cause of cancer death and is increasing in patients under the age of 50 [1]. The incidence in patients aged 20 to 34 years is estimated to increase by 90% by 2030 [2,3]. Rates in younger patients have steadily increased over the last few decades, with trends in the teenaged population also suggesting an increase. 

There are multiple histologic types of colorectal cancer, with the majority categorized as carcinoma. Histologic subtypes have varying characteristics and prognostic associations. Signet-ring cell carcinoma (SRCC) carries one of the poorest prognoses. It is an aggressive cancer subtype with distinct molecular and tumor biology. Tumors are categorized as signet-cell when intracellular mucin displaces tumor cell nuclei in more than 50% of cells within the tumor. SRCC is associated with extensive intramural spread and aggressive behavior leading to carcinomatosis. SRCC therefore typically presents at an advanced stage, contributing to its poor prognosis. 

SRCC is very rare and comprises only 1% of colorectal cancers [4]. It is most commonly diagnosed in patients around age 40. Rarer still is the young adult or teenaged patient. Currently, a very limited number of SRCC cases in young patients have been published. We present a case of a teenaged male with stage IVB signet-ring cell adenocarcinoma of the colon.

## Case presentation

**Figure 1:**
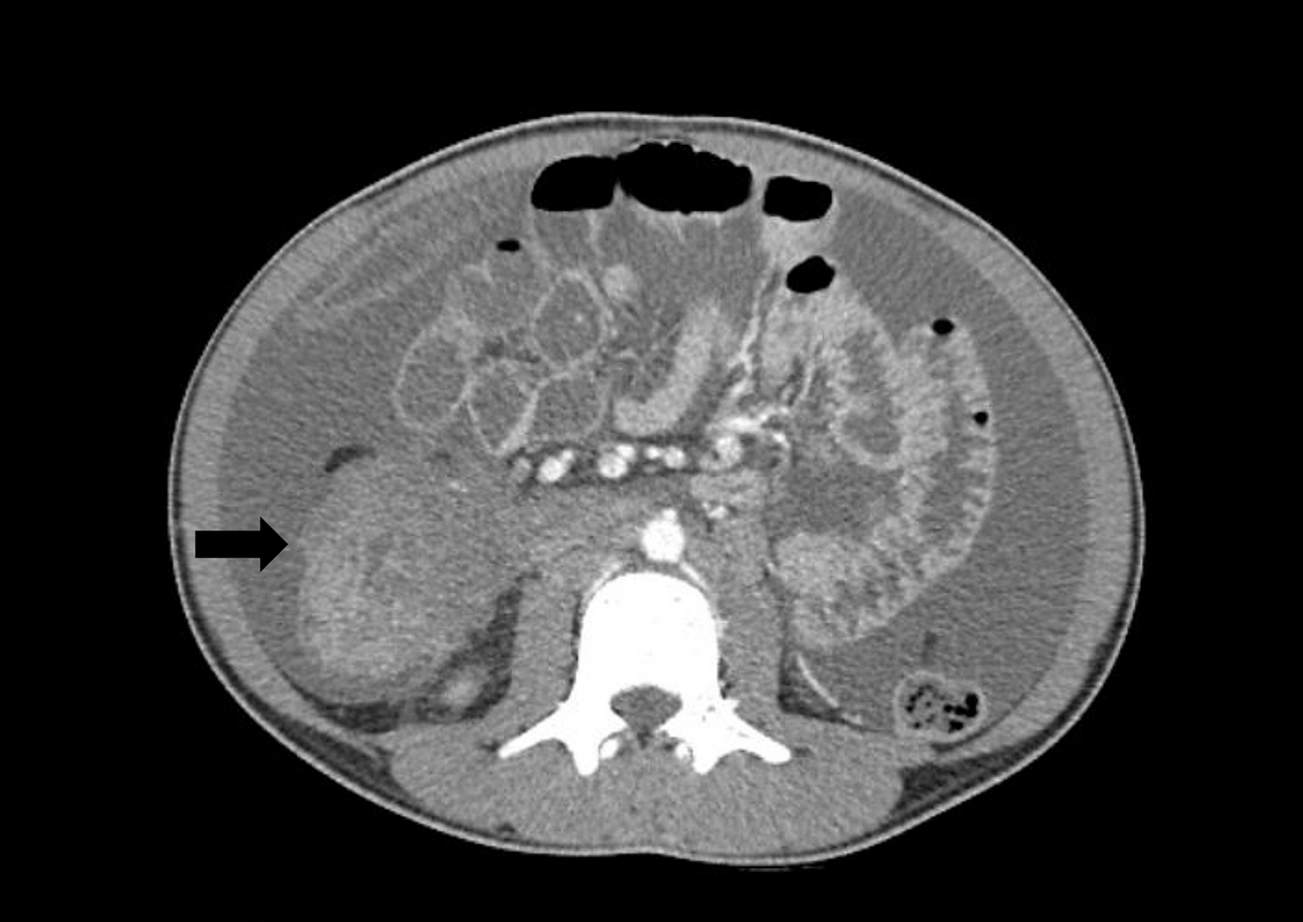
CT scan in axial view demonstrating ascites and a large ascending colon mass (arrow).

An 18-year-old otherwise healthy Arabic male presented to the Emergency Department with one week of vague abdominal pain. Abdominal X-ray and laboratory values were unremarkable. The patient was diagnosed with constipation and discharged home. He returned three weeks later with progressively worsening abdominal pain, distention, and loose stools. On exam, the patient was distended and tender diffusely. A CT scan was obtained which demonstrated ascites and significant thickening of the right colon, concerning for neoplasm (Figure 1).

Laboratory values were within normal limits. Family history was negative for any type of malignancy. The patient was admitted to the hospital and underwent colonoscopy. A friable, ulcerated, and circumferential tumor was encountered in the proximal ascending colon, preventing intubation of the cecum. Given the near-obstruction, the patient was taken to the operating room for right hemicolectomy.

**Figure 2:**
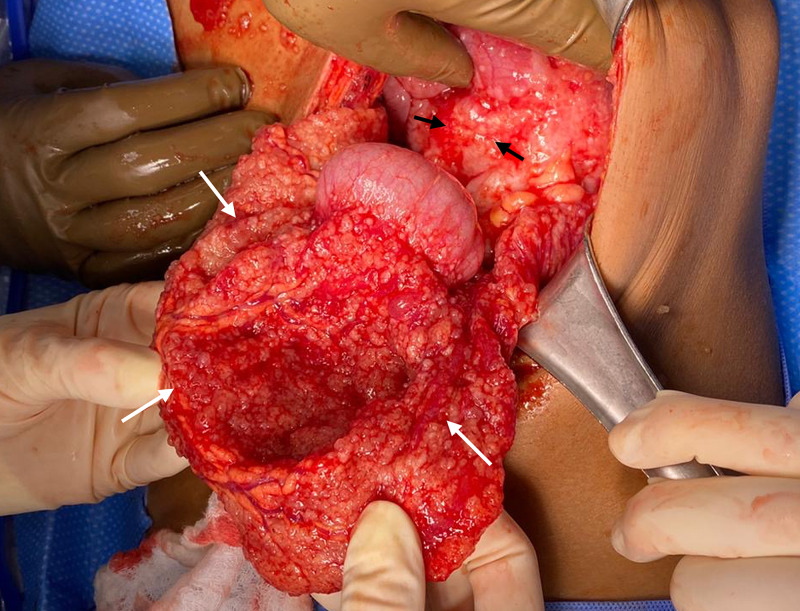
Operative photograph demonstrating metastatic studding throughout the omentum (white arrows) and retroperitoneal tumor involvement (black arrows).

At the operation, 3.5 liters of ascites was evacuated and diffuse carcinomatosis was encountered. Omental caking and metastatic implants throughout the peritoneum and viscera were present (Figure 2). Tumor involvement was noted in the retroperitoneum, inferior vena cava, porta hepatis, gallbladder, and mesocolon. Dense tumor burden prevented any access to the retroperitoneal plane. The decision was made to create a diverting small bowel ostomy as resection of the colon was not possible. The distal ileum was heavily involved, however, a more proximal segment was mobile and free of disease. A portion of the mid-ileum was utilized for a palliative loop ileostomy. Tissue samples were collected and the operation was terminated.

Pathology of omental biopsies and peritoneal fluid demonstrated mucinous adenocarcinoma with many signet-ring cells. The patient was diagnosed with SRCC of colonic origin. Microsatelite instability (MSI) and immunohistochemistry (IHC) testing were negative for Lynch Syndrome, thereby ruling out an inherited defect. 

Subsequent CT of the chest demonstrated extensive metastatic disease in the visualized upper abdomen in addition to a small pulmonary embolism. The patient’s initial CT, obtained three weeks prior to this, was without evidence of any metastatic disease. Carcinoembryonic antigen (CEA) was elevated at 21.5 post-operatively. The patient underwent injectable port placement with plans for FOLFOXIRI chemotherapy to treat stage IVB signet-ring cell adenocarcinoma.

## Discussion

The median age of colon cancer is 68 for males and 72 for females [1]. Despite overall decreasing rates of colorectal cancer, the incidence is rising in the younger population. Still, colon cancer remains uncommon in the young adult and is frequently associated with inherited colorectal conditions or inflammatory bowel disease. The significant rarity of colorectal cancer in the teenaged patient, especially in the absence of risk factors, likely contributes to delays in diagnosis. 

SRCC of the colon is very rare and presents at a younger age compared to non-signet cell adenocarcinoma [5]. The majority of publications regarding SRCC demonstrate an average diagnostic age of 40. Of colorectal SRCC, the majority are rectal in origin. There are few cases of colonic signet-ring cell carcinoma, with fewer than 20 cases in the literature [6]. Rarer still is SRCC of the colon in the teenaged patient.

More than 96% of SRCC cases originate in the stomach. The remaining cases include gallbladder, breast, pancreas, bladder, and colorectal origin. Colonic origin is estimated in less than 1% of cases. SRCC is aggressive and frequently presents at an advanced stage. Median overall survival is between 20-45 months [4]. Age and sex do not appear to affect prognosis. Studies have found that more than 90% of patients are diagnosed at stages III or IV, with more than 50% having peritoneal metastases at the time of diagnosis [4]. 

The greatest prognostic factor for SRCC of the colon is stage at presentation. Therefore, early diagnosis is crucial. Carcinomatosis is much more common in mucinous types of colorectal cancer, like SRCC. Carcinomatosis is also more common with right-sided tumors, consistent with our patient. There is a strong association with Lynch Syndrome, which was not the case in our patient. The patient described had no known risk factors and a negative family history. The rarity of these cases, potentially contributing to delayed diagnosis, in combination with aggressive behavior, lead to dismal outcomes and prognosis. 

## Conclusions

We report a case of signet-ring cell adenocarcinoma of colonic origin in a teenaged patient. This diagnosis is very rare, especially in such a young population. SRCC is aggressive with a poor prognosis. Any delay in diagnosis is significant as this cancer is frequently associated with peritoneal metastases at presentation.
